# Contrast Media: Are There Differences in Nephrotoxicity among Contrast Media?

**DOI:** 10.1155/2014/934947

**Published:** 2014-01-22

**Authors:** Richard Solomon

**Affiliations:** University of Vermont College of Medicine, Burlington, VT 05401, USA

## Abstract

Iodinated contrast agents are usually classified based upon their osmolality—high, low, and isosmolar. Iodinated contrast agents are also nephrotoxic in some but not all patients resulting in loss of glomerular filtration rate. Over the past 30 years, nephrotoxicity has been linked to osmolality although the precise mechanism underlying such a link has been elusive. Improvements in our understanding of the pathogenesis of nephrotoxicity and prospective randomized clinical trials have attempted to further explore the relationship between osmolality and nephrotoxicity. In this review, the basis for our current understanding that there are little if any differences in nephrotoxic potential between low and isosmolar contrast media will be detailed using data from clinical studies.

## 1. Introduction

Radiographic contrast agents have been in use for over 60 years. Based upon current data, 2.0 million cardiac catheterizations are performed annually in the USA [1] and nearly 30 million contrast-enhanced CT scans, in addition to the use of contrast for peripheral angiography. An average contrast-enhanced CT uses ~40 grams of iodine chemically bound to an organic molecule that is injected directly into the vascular system. This is a very large dose of foreign material and speaks to the overall safety of these agents.

Over the past decades, modifications in the organic molecule to which the iodine is bound have occurred and have resulted in changes in the physical properties of the product. These physical properties—ionicity, osmolality, and viscosity—are directly related to the number and size of the organic molecules needed to bind the iodine.

In the mid-1950s, it was recognized that some patients develop a rise in serum creatinine (the definition of contrast-induced acute kidney injury or CIAKI) following the administration of high-osmolar contrast media. Most of these patients received intra-arterial infusions, for example, for coronary angiography. The development of CIAKI has been widely documented in the literature and the risk factors for developing such an injury are exhaustively described [2]. It should be noted that these were observational retrospective studies and no control groups were assessed. Furthermore, no adjudication for other potential causes of a rise in serum creatinine was provided. Nevertheless, animal studies and in vitro studies with lines of kidney cells have provided the basis for the conclusion that contrast media can cause kidney injury. We have come to know much about the pathogenesis of this injury; it involves direct tubule cell nephrotoxicity as well as ischemia mediated by decreased availability of NO and the generation of reactive oxygen species particularly in the medulla of the kidney [3]. In this review, the relevance of the physical properties of the contrast media—ionicity, osmolality, and viscosity—to the development of CIAKI will be reviewed.

## 2. Contrast Media Structure

**Figure 1:**
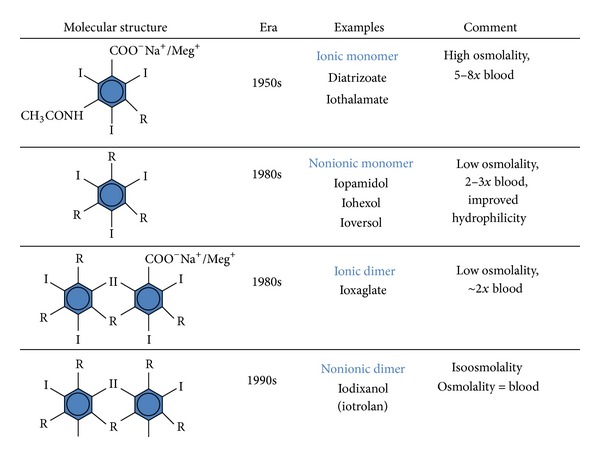
The structure of iodinated contrast media. High-osmolality contrast media (HOCM) have an iodine to molecule ratio of 1.5 : 1. Low-osmolality, nonionic contrast media (LOCM) have an iodine to molecule ratio of 3 : 1. Isosmolar (isoosmolality) contrast media (IOCM) have an iodine to molecule ratio of 6 : 1.

As noted above, the size and number of organic molecules binding the iodine are the primary determinant of the ionicity, osmolality, and viscosity of the commercial product. Over the past 2 decades, we have moved in the development of contrast media from ionic monomers through nonionic monomers to nonionic dimers increasing the number of iodine atoms per molecule from 1.5 to 6.0 (see Figure 1). These changes have resulted in less total molecules (lower osmolality) necessary to deliver a sufficient amount of iodine for adequate imaging but progressively larger molecules (more viscosity) (see Table 1).

Before delving into the multiple studies trying to address the relative nephrotoxicity of the contrast media with different physical properties, it is appropriate to first identify whether any of these differences in physical properties translates into something measureable in *all* patients who receive them.

## 3. Urine Output

In general, the higher the osmolality of the contrast media, the greater the urine output in the first few hours following their administration. This makes sense physiologically as the contrast medium is a nonreabsorbable solute which acts like an osmotic diuretic, reducing electrolyte reabsorption along the nephron and obligating an increase in urine output. An increase in urine output has been observed when comparing high and low-osmolality contrast media as well as low and isoosmolality contrast media [4].

## 4. Urine Viscosity

A definite increase in urine viscosity is observed when using contrast medium that is of very large molecular size (e.g., nonionic dimers). This occurs as a result of the progressive concentration of the contrast medium as it travels down the nephron and 99% of the filtered water is reabsorbed. Urine viscosity can be directly measured and increases significantly only with isosmolar contrast media [4, 5].

Could either of these effects contribute in some way to the decrease in glomerular filtration rate (CIAKI) observed in some patients following contrast administration?

The osmotic diuresis with high- and low-osmolality contrast media *could* result in extracellular volume contraction, stimulation of the intrarenal renin-angiotensin-aldosterone axis, and/or release of other vasoconstrictor hormones (endothelin or adenosine, e.g.) that would augment the direct vasoconstrictor effects of contrast media and further exacerbate ischemia. The increase in urine viscosity *could* raise the pressure within the tubule lumen and reduce the net driving force for glomerular filtration (capillary hydrostatic pressure—(pressure within tubule + plasma oncotic pressure)) and thus directly decrease GFR. The degree to which these predictable effects contribute to the development of CIAKI will depend upon the amount of contrast medium administered and the unique physiologic environment of each patient. For example, the number of nephrons filtering the contrast media (reduced in chronic kidney disease) and the degree of reabsorption within the nephron (stimulated in extracellular volume depletion and congestive heart failure) might be expected to interact with these physiologic effects. As an example, when there is more reabsorption of filtrate because of volume depletion or heart failure, the viscosity in the urine following administration of a nonionic dimer would be greater. Similarly, if a patient is already volume depleted and receives a high-osmolality agent with subsequent increase in urine output, extracellular volume depletion would be exacerbated resulting in a greater stimulation of intrarenal vasoconstrictor factors.

## 5. Defining Nephrotoxicity

To date, the vast majority of clinical trials have used serum creatinine, a marker of glomerular filtration rate, to define CIAKI. The underlying assumption is that with injury to the tubules, there is a reduction in GFR mediated by a variety of mechanisms within the kidney. However, there are many problems with using creatinine as a measure of an acute change in GFR including the following.

- Creatinine is not an ideal marker of GFR because it undergoes secretion and even reabsorption. Changes in serum creatinine could therefore reflect changes in secretion and/or reabsorption without a change in glomerular filtration [6].
- There is a lag phase of 1-2 days before creatinine will rise sufficiently to meet the threshold for CIAKI even when GFR is severely affected. This is because the retained creatinine must distribute in total body water resulting in a slow rise in serum levels. Because of the time lag, an acute reduction in GFR can be missed if serial creatinine levels are not determined for at least 72 hours [7].
- A true change in GFR might occur as a result of hemodynamic changes without any evidence of tubule injury. In this circumstance, an increase in serum creatinine sufficient to define CIAKI would not be associated with true injury.
- Likewise, tubule injury may not always be reflected in a change in GFR. In this circumstance, injury would be missed by reliance on serum creatinine [8].

## 6. Clinical Studies regarding Nephrotoxicity

In the 1980–1990s, the development of nonionic contrast media (low-osmolality contrast medium, ratio: 3 iodines per molecule) was quickly adopted because of a decrease in nonkidney side effects. In particular, the feelings of warmth, nausea, and itching were all diminished with low-osmolality contrast media (LOCM) compared to high-osmolality contrast media (HOCM). Many randomized controlled trials were performed to evaluate whether LOCM reduced the incidence of nephrotoxicity compared to HOCM. An important milestone was the meta-analysis of these trials performed by Barrett and Carlisle in 1993 [9]. These authors indeed found a reduction in the incidence of CIAKI with the use of a low- compared to the high-osmolality contrast media. However, a more in-depth review of their publication reveals the following.

- There was no difference in the incidence of CIAKI in patients who had normal kidney function.
- There was a 50% reduction in the incidence of CIAKI only in patients with an initial creatinine clearance less than 60 mL/min.
- The reduction in the incidence of CIAKI was seen only when the contrast was given intra-arterially but not intravenously.

Despite these findings, this meta-analysis contributed significantly to the disuse of HOCM in all patients and HOCM subsequently faded from the market to be replaced with LOCM.

In the late 1990s, a new class of contrast agent—isosmolar or IOCM—came into clinical use. Based upon the meta-analysis of Barrett and Carlisle that found that osmolality contributed to the nephrotoxicity of contrast media under certain circumstances, it was quite reasonable to hypothesize that the IOCM would be associated with less CIAKI compared to LOCM. A seminal study supporting this claim was the NEPHRIC trial. This was a 129-patient, randomized trial in patients with diabetes and chronic kidney disease (estimated creatinine clearance <60 mL/min) who were undergoing cardiac angiography [10]. The IOCM (iodixanol, Visipaque) was compared to a LOCM (iohexol, Omnipaque) with the primary endpoint of CIAKI defined as a >0.5 mg/dL absolute rise in creatinine over 48–72 hours. The incidence of CIAKI with iodixanol was significantly lower than with iohexol (2% versus 16%, resp.) in this high-risk population.

**Figure 2:**
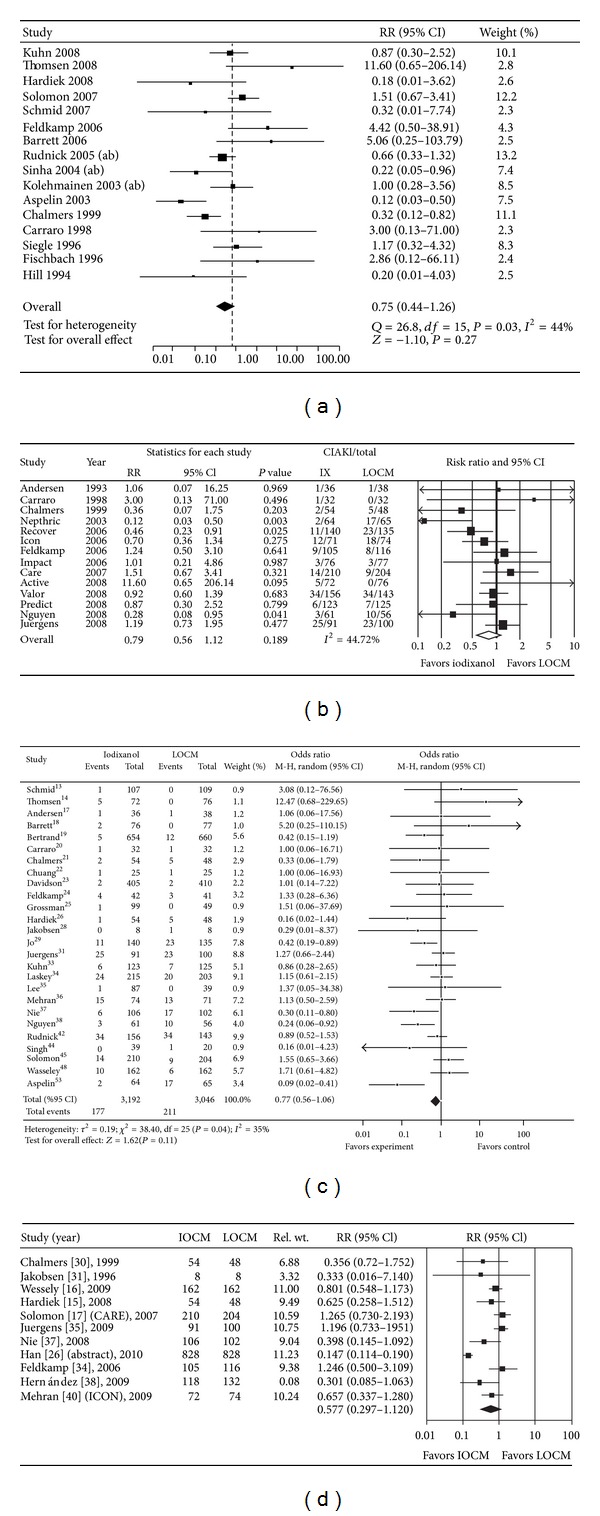
Meta-analyses of randomized prospective trials comparing LOCM to IOCM. (a) Heinrich et al., 16 trials; published and abstracts, IV and IA contrast, through 2007. (b) Reed et al., 14 trials; published data, IV and IA contrast, through 2008. (c) From et al., 26 trials; IV and IA contrast, through 2009. (d) McCullough and Brown, 11 trials, only IA contrast, published and abstracts, through 2010.

What followed the publication of the NEPHRIC trial in the New England Journal of Medicine in 2003 can aptly be described as a “war.” Manufacturers of LOCM other than the one studied in the NEPHRIC trial immediately set to work to replicate or repudiate the finding using their own LOCM. Multiple RCTs were conducted in high-risk populations undergoing cardiac angiography, peripheral angiography, or contrast-enhanced CT. Over the past decade, at least 4 meta-analyses of these trials were published with essentially similar findings [11–14] (see Figures 2(a), 2(b), 2(c), and 2(d)). Some meta-analyses mixed intravenous and intra-arterial administration, while others excluded studies presented in abstract. Most meta-analyses found little evidence of heterogeneity strengthening the conclusions of the analyses. The incidence of CIAKI (by any definition) was similar between LOCM and IOCM with the possible exception of the LOCM, iohexol [12].

Was NEPHRIC just wrong? It is always easy to argue that a study with only 129 subjects and in which the primary endpoint occurs in <10% is going to be underpowered and subject to a type-1 error. This is certainly one possibility. Was NEPHRIC correct but used as a comparator a particular LOCM (iohexol) with a higher with a higher incidence of CIAKI? The subsequent studies using other LOCMs would support this conclusion as well. If one refers to Table 1, iohexol has the highest viscosity of the LOCMs. Perhaps, viscosity and osmolality both contribute to nephrotoxicity (see above). To date, there has not been a randomized controlled trial comparing iohexol to any other LOCM in a high-risk population. The updated 2011 AHA/ACC guidelines for use of contrast media in high-risk patients do not favor IOCM over LOCM [15].

Thus, an overall summary of the current database, updated since previous guideline recommendations, is that strength and consistency of relationships between specific isosmolar or low-osmolar agents and CIAKI or renal failure are not sufficient to enable a guideline statement on selection among commonly used low-osmolar and isosmolar media.

What are the limitations of these studies and the subsequent meta-analyses? These randomized prospective trials all used serum creatinine as the marker of injury. They assumed that any increase in creatinine following contrast exposure was caused by the contrast (no adjudication for other causes of a rise in serum creatinine). Katzberg et al. noted persistent global and focal nephrograms on CT performed 24 hours following administration of isosmolar contrast for coronary angiography [17]. Such findings might be explained by microemboli rather than contrast-induced nephrotoxicity. Unfortunately, the study did not measure either serum creatinine or injury markers raising the possibility that the findings are simply a paraphenomenon. Indeed others have noted similar persistent nephrograms following isosmolar contrast and suggested that increased viscosity of isosmolar contrast leads to longer retention times within the kidney [18]. Regardless of mechanism, the findings do not exclude the possibility of direct nephrotoxicity from contrast.

## 7. Intravenous versus Intra-Arterial Injections

What about differences between contrast media when given by the intravenous route? There is a significant body of literature suggesting an overall lower incidence of nephrotoxicity when iodinated contrast is given intravenously compared to intraarterially [19]. The basis for this difference in nephrotoxicity is multifactorial and includes the following:

- differences in chronic comorbidities between those getting IV versus IA contrast media,
- differences in dose of contrast administered,
- ascertainment bias resulting from different follow-up protocols in those receiving IA versus IV contrast,
- bias resulting from acute hemodynamic instability, more likely in hospitalized versus nonhospitalized patients and thus more likely in IA versus IV contrast administration.

**Figure 3:**
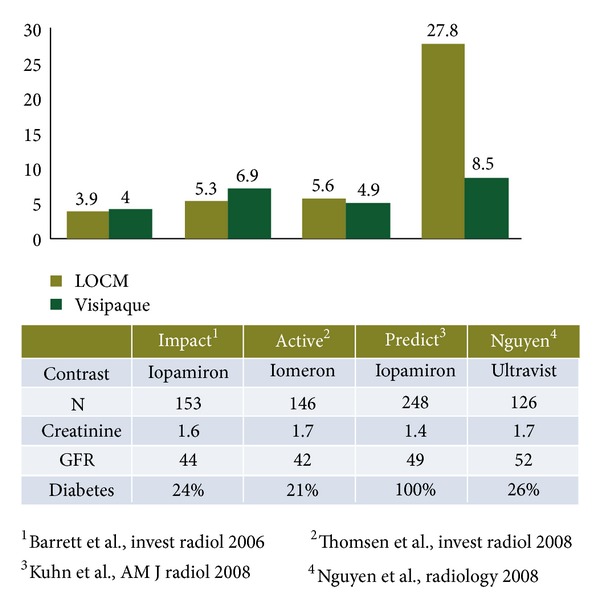
Randomized prospective trials of intravenous-only IOCM versus LOCM.

When prospective randomized trials have specifically addressed patients undergoing contrast-enhanced CT, no differences have been found between IOCM and LOCM (Figure 3). A single exception was a trial by Nguyen, the smallest of the studies that is limited to a single center.

However, it should again be emphasized that the development of a rise in serum creatinine following a contrast-enhanced CT may not reflect kidney injury or specifically injury resulting from contrast (see above regarding limitation of using serum creatinine). Randomized controlled trials using specific injury markers that are known to increase after contrast induced injury. Even with injury markers such as NGAL, IL-18, and KIM-1, there is no specificity for contrast-induced injury.

In addition, a number of authors have found that the incidence of a rise in serum creatinine following a non-contrast-enhanced CT is similar to that following a contrast-enhanced CT, particularly in those without severe reduction in GFR at baseline [20–22]. This suggests that other explanations for a rise in creatinine in these patients should be explored. The most likely explanation would be hemodynamic instability in patients with an indication for a contrast-enhanced CT. Interestingly, a recent abstract found no evidence for an increase in urinary markers of injury (KIM-1 and NGAL) following 511 contrast-enhanced CT exams [23]. In this population, 3.9% developed CIAKI by a usual definition using serum creatinine changes.

## 8. Other Acute Endpoints

A few studies have focused on the thrombogenicity of different contrast agents. In vitro studies have suggested that ionic low-osmolar contrast is less thrombogenic compared to either isosmolar or low-osmolar nonionic contrast [24]. Clinical trials evaluating potential thrombotic complications have focused on MACE (cardiac death, recurrent nonfatal acute MI, and emergency CABG or repeat PCI) or acute and subacute stent restenosis [25, 26]. Both trials reported a significant reduction in the primary endpoints with ionic low-osmolar contrast. These events occur infrequently which leads to concerns about the power of the individual studies. They were also done during an era when anticoagulation protocols were different than current protocols. Finally a number of other studies could not replicate these results so the issue remains unsettled [27–30].

## 9. Long-Term Outcomes

While the meta-analysis data seems clear regarding an absence of differences in nephrotoxicity between IOCM and LOCM, there is relatively little data regarding long-term consequences following contrast administration. Since the contrast is eliminated from the body almost completely within 24 hours, any long-term consequences related to contrast administration are likely related to acute kidney injury at the time of administration, for example, nephrotoxicity or the comorbidities present in patients who developed acute kidney injury. Again, the assumption has been that the acute kidney injury is related to the contrast and not some other mechanism. Many observational studies and retrospective reviews of databases have reported on the association between CIAKI and congestive heart failure, mortality, and the long-term loss of kidney function including the need for dialysis. It is beyond the scope of this review to discuss whether this association represents causality or not. The reader is referred to reviews of this topic [31].

Reed et al. [14] in a meta-analysis of 16 trials involving 2763 patients found no differences in postprocedure death or need for dialysis between IOCM and LOCM. However, data was not provided in all studies and very few events actually occurred (a total of 11 and 12 events, resp.).

In an observational study by Per Liss and collaborators, the risk of readmission to the hospital with a diagnosis of acute kidney injury within 1 year following the exposure to an isosmolar or low-osmolar contrast media is described using data from the Swedish Cardiovascular Registry [32]. The Swedish hospital system mandates use of only one contrast agent in each hospital. These authors found a higher incidence of acute kidney injury readmission in hospitals using an IOCM compared to any LOCM. Furthermore, in hospitals that switched from IOCM to LOCM during the years of the study, the incidence of acute kidney injury decreased. Since this is an observational database and not a randomized trial, the results should be taken with caution. However, the uniqueness of the Swedish system eliminates many sources of bias inherent in other observational databases.

The CARE follow-up trial followed patients for one year who were enrolled in a prospective randomized trial comparing iopamidol (low-osmolar contrast media) to iodixanol (isosmolar contrast media) [16]. Prespecified endpoints included death, stroke, myocardial infarction, end-stage kidney disease, percutaneous coronary revascularization, coronary artery bypass graft surgery, other revascularization procedures (e.g., carotid, runoff vessels), and others (e.g., cardiac arrest, development of congestive heart failure or pulmonary edema, and need for permanent pacing). Four events were considered major adverse events: death, stroke, myocardial infarction, and ESRD. When more than one event occurred in the same patient, only the first event was used for analysis. In this study, CIAKI was defined using a number of novel markers: an absolute increase in serum creatinine of 0.3 mg/dL and a 15%, 20%, or 25% increase in serum Cystatin C (to avoid some of the problems mentioned above regarding creatinine). All of these novel definitions were associated with a 2-fold increase in the incidence of adverse events. There were no differences in baseline risk factors, demographics, or procedural characteristics between the two groups. At one year, there was a statistically significant difference in all adverse events and major adverse events in favor of iopamidol. This reduction in adverse events was associated with a reduction in the incidence of CIAKI in the iopamidol group using any of the novel AKI markers.

## 10. Conclusions

Contrast media are extremely safe but can precipitate acute kidney injury in a small number of high-risk patients. Based upon randomized trial data, there do not appear to be significant differences in the nephrotoxicity between contrast media that differ on the basis of ionicity, osmolality, or viscosity. This conclusion applies to both the intravenous and intra-arterial administration of contrast. Although a few trials have described long-term differences in outcomes based on viscosity and osmolality, further studies are clearly needed to define potential mechanisms and to confirm these preliminary findings.

## Figures and Tables

**Table 1:** Comparison of physical properties of commonly used iodinated contrast media.

	Concentration (mgl/mL)	Grams of iodine/100 mL	Osmolality (mmol/Kg at 37°C)	Viscosity (cP at 37°C)
Iodixanol (Visipaque)	270–320	27–32	290	6.3–11.8
Iohexol (Omnipaque)	140–350	14–35	322–844	1.5–10.4
Iopamidol (Isovue)	200–370	20–37	413–796	2.0–9.4
Iopromide (Ultravist)	150–370	15–37	328–774	1.5–10.0
Ioversol (Optiray)	160–350	16–35	355–792	1.9–9.0
